# Monoclonality and Low Genetic Diversity in *Vanilla shenzhenica*: Highlighting Urgent Need for Genetic Preservation of China’s Only Endangered *Vanilla*

**DOI:** 10.3390/ijms26073451

**Published:** 2025-04-07

**Authors:** Li Xiao, Ai-Qun Hu, Mei-Na Wang, Zhuo Cheng, Kuan-Bo Chi, Chun-Lin Long, Jin-Gang Liu

**Affiliations:** 1 College of Life and Environmental Sciences, Minzu University of China, Beijing 100081, China; xl990828@163.com (L.X.); 18515952092@163.com (Z.C.); 2 Shenzhen Key Laboratory for Orchid Conservation and Utilization, the National Orchid Conservation Center of China and the Orchid Conservation & Research Center of Shenzhen, Shenzhen 518114, China; daisyhu2012@gmail.com (A.-Q.H.); snow-wmn2005@163.com (M.-N.W.); ckb2129@163.com (K.-B.C.); 3 Key Laboratory of National Forestry and Grassland Administration for Orchid Conservation and Utilization, The Orchid Conservation & Research Center of Shenzhen, Shenzhen 518114, China; 4 Key Laboratory of Ecology and Environment in Minority Areas, Minzu University of China, National Ethnic Affairs Commission, Beijing 100081, China; 5 Key Laboratory of Ethnomedicine, Minzu University of China, Ministry of Education, Beijing 100081, China; 6 Institute of National Security Studies, Minzu University of China, Beijing 100081, China

**Keywords:** clonality, ddRAD-seq, endangered species, genetic diversity, species conservation, *Vanilla*

## Abstract

Long-term clonality has profound consequences for genetic structure despite offering an alternative means of reproductive assurance under unfavorable conditions for sexual reproduction. *Vanilla shenzhenica* Z. J. Liu & S. C. Chen (Orchidaceae), the only endangered *Vanilla* species in China, exhibits a clear tendency towards asexual propagation, as evidenced by its small, fragmented wild populations. To develop effective conservation strategies for this species, it is essential to assess the extent of clonality and evaluate genetic diversity both within and among populations. In this study, we sampled 43 individuals from cultivated and wild populations of *V. shenzhenica* and analyzed their phylogenetic relationships, genetic structure, and diversity based on single-nucleotide polymorphisms (SNPs). Our results indicate that all the studied wild populations are predominantly sustained by vegetative growth, each forming a monoclonal patch with a single genotype. The overall genetic diversity within *V. shenzhenica* is low likely due to a combination of factors, including clonality, reduced effective population size, and environmental disturbances. These findings underscore the urgent need for the conservation management of this species. Conservation plans should prioritize ex situ conservation efforts, focusing on promoting assisted sexual reproduction to produce viable seeds and offspring that combine diverse genotypes from different populations. This study provides valuable insights in relation to effective conservation planning for endangered clonal species.

## 1. Introduction

Flowering plants display the widest range of strategies for achieving both sexual and asexual reproduction [1]. Clonal propagation, which offers numerous ecological advantages [2,3,4], may provide an alternative method for reproductive assurance in many species that face unfavorable environmental conditions for sexual reproduction. This strategy can enable species to persist for extended periods, even after habitat fragmentation, and make them more tolerant of various extinction events [5].

Despite the many benefits of clonal reproduction, there is growing recognition that clonal growth can negatively impact sexual reproduction by limiting resource allocation to flowering and seed production, interfering with pollination and mating, and causing the accumulation of somatic mutations that reduce fertility [6]. In addition, vegetative propagation can influence a population’s genetic structure and genotype diversity [7]. A direct consequence of prolonged clonal reproduction and the suppression of sexual reproduction is that the clones that are less locally adapted are outcompeted by the expanding ramets of more-adapted genotypes, ultimately leading to monoclonal patches [8]. In a population dominated by vegetative propagation, a single clone (genet) can gradually produce many individuals (ramets), resulting in an apparently large population size with limited genetic diversity or even a single genotype. This can be misleading by suggesting a large and healthy population, thereby overestimating the effective population size. As a consequence, such a situation may lead to an underestimation of the species’ risk of endangerment, potentially causing people to misjudge its conservation status and miss critical opportunities for preservation, particularly when vulnerable populations face catastrophic events or habitat degradation [9]. Accordingly, it is essential to understand the extent of clonality, especially when managing the conservation efforts for endangered clonal species. Accurately identifying clonality can be challenging in some plants, particularly when the connections between ramets are complex; therefore, genetic investigations are an ideal approach to this.

**Figure 1:**
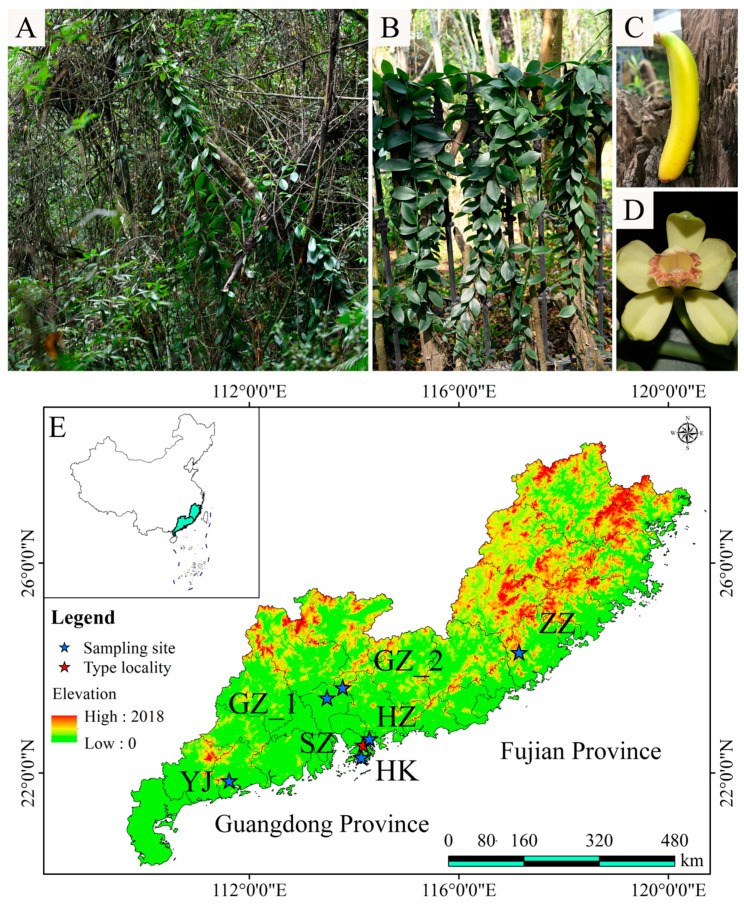
Habitat, morphological characters, and sampling sites of *Vanilla shenzhenica*. (**A**) Wild population in Zhangzhou, Fujian. (**B**) Cultivated plants in China National Orchid Conservation Center (CNOCC) in Shenzhen, Guangdong. (**C**) Fruiting pod in Zhangzhou, Fujian. (**D**) Flower in CNOCC in Shenzhen, Guangdong. (**E**) Geographic distribution of studied populations. Note: different populations are defined as GZ = Guangzhou; YJ = Yangjiang; SZ = Shenzhen; HZ = Huizhou; ZZ = Zhangzhou; HK = Hong Kong.

In most *Vanilla* orchids, sexual reproduction is rarely observed under natural conditions, with vegetative propagation from stem cuttings being the predominant mode of reproduction [10]. *Vanilla shenzhenica* Z. J. Liu & S. C. Chen (Figure 1A,C,D) [11], one of the four *Vanilla* species found in China, is endemic to southern China, mainly in Guangdong and its surrounding areas. As the only *Vanilla* species listed in *China’s National Key Protected Wild Plants* [12], *V. shenzhenica* has small, fragmented populations and quite a narrow distribution. Field surveys have shown that the distribution area of existing populations varies significantly, ranging from less than 100 m^2^ to a maximum of 1000 m^2^. Unfortunately, the wild population at the type locality (Longgang, Shenzhen, China) has disappeared, and no individuals can currently be found. The China National Orchid Conservation Center (CNOCC) in Shenzhen, Guangdong, transplanted some wild individuals from the Huizhou and type locality in Shenzhen between 2007 and 2009; however, with the expansion of cuttings, these individuals were gradually mixed into the population, and their detailed information was lost (Figure 1B). The fruiting seed pods of *V. shenzhenica* are rarely observed, despite the presence of flowering having been noticed in wild populations. Similarly, cultivated individuals seldom produce flowers regularly but instead exhibit vigorous vegetative growth. These phenomena suggest a low rate of sexual reproduction in this species, which is consistent with the general reproductive patterns of the *Vanilla* genus [10]. Therefore, it is important to assess the extent and implications of clonal reproduction in *V. shenzhenica* and to develop appropriate conservation strategies. In this study, using double-digest restriction site-associated sequencing (ddRAD-seq), the specific aims are as follows: (1) to identify the origin of ex situ-conserved wild individuals at CNOCC; (2) to confirm the current mode of reproduction in the wild populations and assess the extent of clonality; (3) to estimate the genetic diversity and examine factors contributing to its endangered status; and (4) to propose conservation measures to guide the effective management of endangered clonal species.

## 2. Results

### 2.1. Sequencing Data, Phylogeny, and Genetic Structure

ddRAD-seq generated a total of 94,996,451,408 paired-end reads for *V. shenzhenica* (n = 43). The average GC content and Q30 score across all samples were 37.67% and 97.56%, respectively. A total of 669,102 raw single-nucleotide polymorphisms (SNPs) were identified using the pipeline in STACKS v.2.54 [13]. In total, 241,871 SNPs were retained for further population analyses after filtering using VCFtools v.0.1.13 [14].

**Figure 2:**
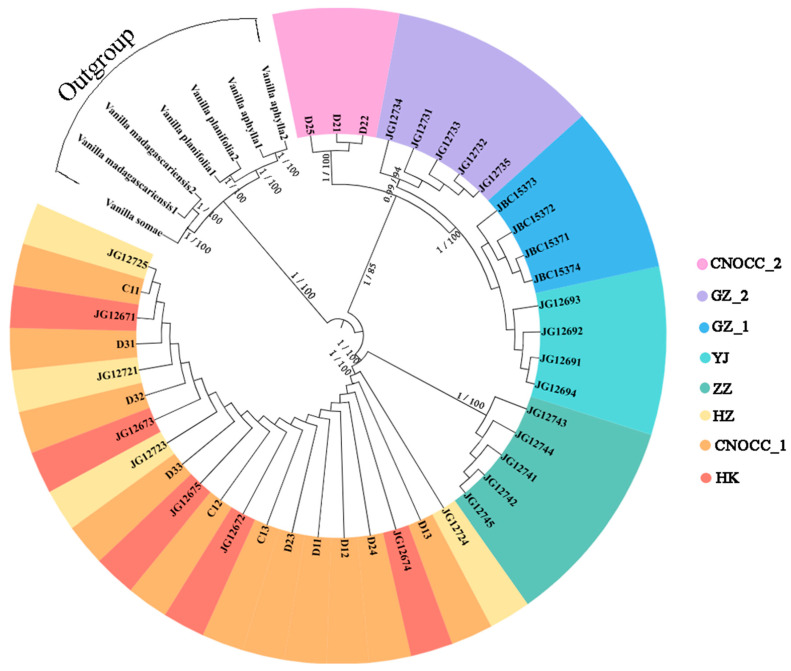
Maximum likelihood (ML) tree and Bayesian inference (BI) tree based on chloroplast genomes (*cp*DNA) of 41 *V. shenzhenica* individuals and outgroups. Different colors represent different populations. Numbers on branches indicate posterior probabilities (PP ≥ 90) and bootstrap support (BS > 50). From left to right: BI posterior probabilities/ML bootstrap support. Different colors represent individuals of *V. shenzhenica* from different populations.

A total of 41 complete chloroplast genomes (*cp*DNA) were successfully assembled for phylogenetic reconstruction. The best-fitting model for phylogenetic tree construction was GTR + F + G4. The phylogenetic tree based on *cp*DNA (Figure 2) revealed that the GZ_1, GZ_2, and YJ populations were confirmed to belong to *V. shenzhenica*. The HZ and HK populations, when grouped together, exhibit a closer phylogenetic relationship. Most transplanted individuals at CNOCC, especially CNOCC_1, were likely allied with the HZ population, suggesting they were originally transplanted from Huizhou. Notably, individuals D21, D22, and D25 from CNOCC_2 were significantly separated from CNOCC_1, which indicates that these three individuals may have originated from the type locality in Shenzhen.

**Figure 3:**
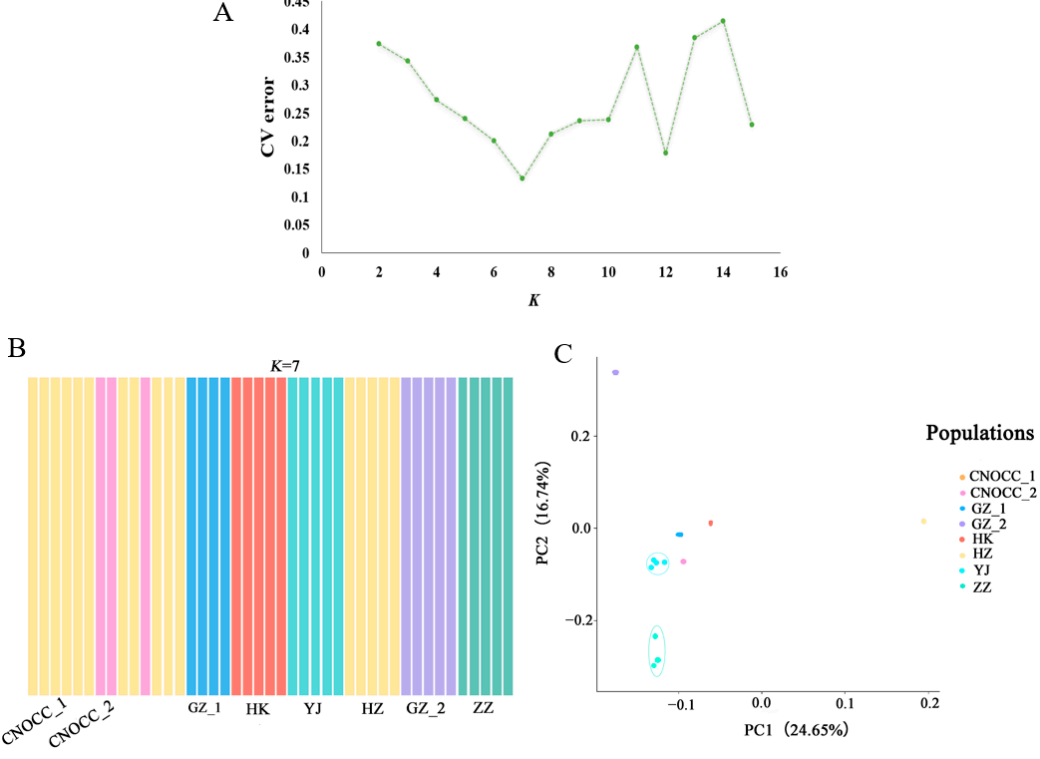
The genetic structure of *V. shenzhenica* based on SNPs. (**A**) The ADMIXTURE cross-validation (CV) error rate corresponding to the different number of substructures (*K*) values ranging from 2 to 15, with *K* = 7 showing the smallest cross-validation (CV) error. (**B**) The population structure based on ADMIXTURE analysis with *K* = 7 (i.e., the optimal solution). Different colors represent different genetic clusters. (**C**) Principal component analysis (PCA). The percent variation explained by each component is shown in parentheses; each sample is represented by a point, and points of CNOCC_2 and points of HZ have overlapped according to the data.

**Figure 4:**
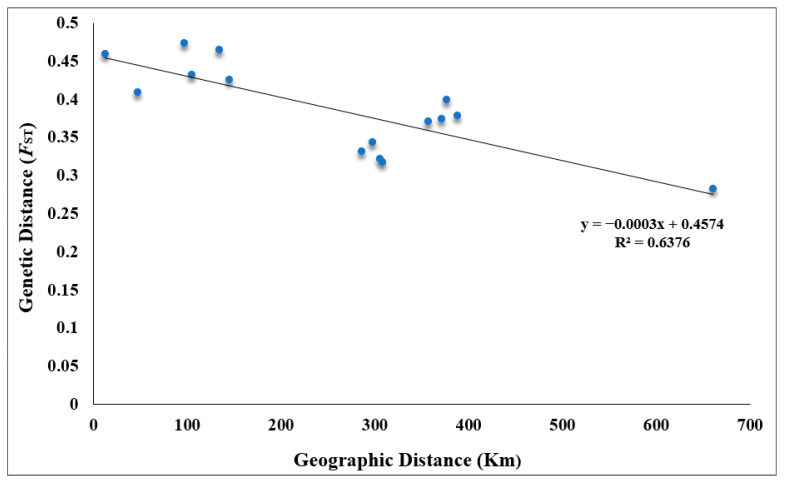
The genetic and geographic distance of the V. shenzhenica populations based on the Mantel test (*p* = 0.952). *F*_ST_ fixation index.

The minimum cross-validation (CV) error reached its lowest point when it was assumed that the number of substructures (*K*) was seven (Figure 3A), suggesting the division of all individuals into seven genetic clusters across all populations. Consistent with the phylogenetic analysis, the structure analysis also indicated that CNOCC_1 originated from Huizhou, exhibiting an identical genetic structure to the HZ population. Meanwhile, CNOCC_2 showed a different structure from the others, which may indicate that they belong to the population from the type locality in Shenzhen. The structure analysis revealed a clear genetic division, with each population being well separated from each other; however, within the groups, there was generally a high genetic homogeneity. The principal component analysis (PCA) confirmed the results of the population structure analysis, demonstrating that populations of *V. shenzhenica* are highly different from each other (Figure 3C). Additionally, the genetic clusters corresponded to geographic localities; however, the Mantel test showed no significant correlation between genetic distance and geographic distance (*p* > 0.05; Figure 4).

### 2.2. Genetic Diversity

Based on the results from the phylogenetic tree and genetic structure analysis, we included individuals of CNOCC_2, as they are representative of the population from the type locality in Shenzhen, for the following analyses. Using SNPs, a statistical analysis of the genetic indices was performed (Table 1). The observed heterozygosity (*H*o) across all populations from 0.172 to 0.219 (average 0.198), and the expected heterozygosity (*H*e) across all populations ranged from 0.094 to 0.165 (average 0.118). The *H*e value for each population was consistently higher than the *H*o value, and the nucleotide diversity (π) across all populations ranged from 0.099 to 0.185 (average 0.132). The negative inbreeding coefficient (*F*_IS_) in each group suggested an excess of heterozygotes across all populations. Based on these *H*e and π values, within-population genetic diversity levels were rank-ordered as YJ > ZZ > CNOCC_2 > GZ_2 > HK > GZ_1 > HZ > CNOCC_1.

Individuals from CNOCC_1 exhibited similar genetic diversity patterns to the HZ population, with a low pairwise fixation index (*F*_ST_) value (0.009; Table 2). These results, along with the findings from the genetic structure analysis and phylogenetic tree, confirmed that cultivated individuals in CNOCC_1 originated from Huizhou. In contrast, pairwise comparisons between other populations revealed high genetic differentiation, with pairwise *F*_ST_ values exceeding 0.25 (Table 2). Among them, a comparative higher degree of differentiation (*F*_ST_ = 0.478) was observed between CNOCC_2 and the HZ population. The YJ and ZZ populations, despite being the most distant geographically, did not exhibit the highest pairwise *F*_ST_ value, which is consistent with the results of the Mantel test (Figure 4).

### 2.3. Historical Population Dynamics

**Figure 5:**
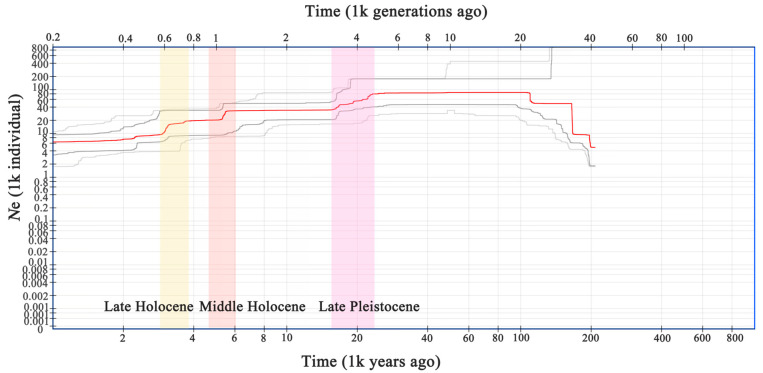
Changes in population size inferred from the Stairway plot using folded site frequency. The red line represents the estimate of the effective population size (*N*e), and the two gray lines depict the 95% confidence interval for the estimate.

The Stairway plot based on SNPs revealed that the effective population size (*N*e) of *V. shenzhenica* began to gradually decline in the late Pleistocene, approximately 28–18 kya (thousand years ago), followed by a period of stability (Figure 5). Subsequently, a sharp decline occurred during the middle Holocene (5–6 kya), indicating a putative population bottleneck. *N*e continued to decrease and experienced another population bottleneck event during the late Holocene (around 3 kya); afterwards, it maintained a relatively stable size, estimated at nearly 6000. Overall, the Stairway plot based on SNPs detected that there were at least three historical declines in the effective population size of *V. shenzhenica*, with *N*e decreasing from 80,000 to 6000.

## 3. Discussion

### 3.1. Clonal Propagation and Ultimately Monoclonal Populations of V. shenzhenica

In summary, the genetic status of *V. shenzhenica* is characterized by high genetic differentiation among populations, relatively low genetic diversity, a lack of inbreeding depression (*F*_IS_ < 0), and an excess of heterozygotes within populations (*H*o > *H*e). In particular, the heterozygote excess is an uncommon but typical phenomenon in small, outcrossing populations that exhibit self-incompatibility among individuals and rely on vegetative growth [1,15]. This pattern can be observed in many species where sexual reproduction is absent and clonal propagation predominates, as has been reported in *Populus tremuloides* [16], *Laminaria rodriguezii* [17], *Melaleuca deanei* [18], and *Prunus avium* [15]. As demonstrated by Stoeckel et al. [15], heterozygote excess may result from several potential factors, including self-incompatibility mechanisms, asexual reproduction, and a small reproductive population size. In this study, *V. shenzhenica*, which is similar to most species of the *Vanilla* genus, currently relies on clonal propagation, as can be seen by the extremely low fertility observed in wild populations.

Most *Vanilla* species are self-incompatible, depending on the pollinator, for sexual propagation [19]. The typical floral structure of *Vanilla* also makes natural self-pollination difficult in most *Vanilla* species, including *V. shenzhenica*, due to a small structure called the rostellum that separates the stigmata and anthers. This barrier prevents self-pollination unless a pollinator or human intervention occurs, except for some rare species like *V. palmarum* [20] and *V. inodora* [20]. In this study, the negative *F*_IS_ values in all populations indicated a lack of inbreeding in *V. shenzhenica*, while populations of asexual or self-incompatible species are typically expected to exhibit negative *F*_IS_ values [1,21]. Furthermore, hand-pollination experiments were conducted at CNOCC on the living plants transplanted from the wild population in Huizhou. We observed that the fruiting seed pods were either aborted or no seeds were found in the mature fruits. Taken together, *V. shenzhenica* is likely to be a species with self-incompatibility, although this remains to be confirmed using an empirical study.

In most cases where populations shift from sexual to asexual reproduction, environmental factors—such as a lack of pollinators, unfavorable conditions for flowering, seed maturation, or seedling establishment—are the primary causes [22,23]. Under these circumstances, the investment of resources into sexual reproduction may yield minimal returns and selection may favor a reduction in the investment, thereby increasing energy allocation to survival and/or clonal propagation [23]. However, a direct consequence of prolonged clonal reproduction and the suppression of sexual reproduction is that local less-adapted clones could be outcompeted by the expanding ramets of more-adapted genotypes, ultimately leading to monoclonal patches [5].

The genetic structure analysis revealed that each wild population of *V. shenzhenica* formed its own unique genetic cluster, with individuals within each cluster sharing an identical genetic structure. Notably, the classification of these genetic clusters corresponded exactly to their geographic origin. However, the Mantel test showed no significant correlation between genetic distance and geographic distance. These findings suggest that the current populations of *V. shenzhenica* may each represent a monoclonal patch with a single genotype. Consequently, it is unsurprising that ex situ-conserved individuals at CNOCC share the same genetic structure as that of the HZ population. A similar circumstance occurred in other orchids—*Bulbophyllum bicolor*—in which 13 out of the 15 populations tested were monoclonal, exhibiting a single genotype [24]. While clonality can provide reproductive assurance under unfavorable conditions for sexual propagation, it also reduces the genetic diversity within populations. In addition, there is a consensus that species undergoing long-term clonality may experience a degeneration of traits associated with sexual reproduction, eventually becoming incapable of reproducing sexually, which is known as the loss of sex [5,23]. This phenomenon has been documented in *Decodon verticillatus* [25] and *B. bicolor* [24]. This trend is particularly evident in species that are self-incompatible or obligate outcrossers. The ultimate consequence of the vicious cycle of decreasing genotype diversity and reduced mate availability is the formation of a monoclonal population, leading to the loss of sexual reproduction [26]. In any case, clonal plants that have lost the ability to sexually reproduce may be less effective at spreading into new habitats and may respond more slowly to habitat changes than nonclonal species, potentially culminating in local extinction [27,28,29].

### 3.2. Genetic Diversity of V. shenzhenica

The evaluation of genetic diversity constitutes a cornerstone in the conservation strategies for endangered species. In this study, we investigated the genetic diversity of remnant populations of *V. shenzhenica*, with an observed species-level *H*e of 0.135 and π of 0.148. These values were lower when compared to those of endangered plants with low genetic diversity assessed identically based on SNPs, including *Phoebe zhennan* (*H*e = 0.145, π = 0.158) [30], *Horsfieldia hainanensis* (*H*e = 0.167, π = 0.172) [31], and *Panax notoginseng* (*H*e = 0.155, π = 0.159) [32]. This is expected, as small, isolated populations often show low genetic variation due to limited gene flow, genetic drift, and inbreeding depression [33]. However, negative *F*is in the populations of *V. shenzhenica* suggested that inbreeding depression did not account for this phenomenon.

Generally, genetic diversity is influenced by multiple factors, including a species’ evolutionary history, geographic range, breeding systems, and seed dispersal mechanisms such as wind or animal ingestion [34]. Here, we discuss the factors contributing to the reduced genetic diversity of *V. shenzhenica*. Long-term clonality with very limited gene flow among populations, along with the irrelevant relation between genetic and geographic distance, which can explain the current status quo of the small, fragmented distribution of populations, contributes to the decrease in genetic diversity [35,36]. In addition, the species’ historical population dynamics were analyzed, considering that smaller population sizes are commonly associated with reduced genetic variation due to genetic drift [33,37,38]. *V. shenzhenica* has experienced at least a three-fold decline in *Ne* throughout its history (Figure 5). Notably, two putative population bottleneck events with a sharp decline in population size were detected during the middle Holocene (5–6 kya) and the late Holocene (around 3 kya). The first population bottleneck may be caused by the sea level rise in the South China Sea, which flooded much of the plains in southern China [39], while the second may be related to a significant climate variation triggered by abrupt changes in tropical–subtropical climate patterns across the Pacific Ocean (3–4 kya) [39]. These putative bottleneck events could have reduced the variation in the gene pool of the populations of *V. shenzhenica*; thereafter, only smaller populations with lower genetic diversity remain to pass on these genetic features to future generations. Additionally, we have documented the extinction of a wild population from the type locality in Shenzhen, which was first published in 2007; however, during our two field surveys conducted in 2019 and 2023, we found this wild population had disappeared. The main cause is likely linked to human activities, as the area is located on a tourist mountain that is frequently visited by hikers. Human disturbance and habitat fragmentation are likely reducing genetic diversity [40].

### 3.3. Conservation Implications

The population structure patterns revealed in our study have important conservation implications. Firstly, this study highlights the urgent need for ex situ conservation, which can effectively preserve genetic resources when wild populations are at risk of extinction [41,42]. Although the wild population from the type locality is now extinct, individuals that were transplanted from this site have been successfully preserved at CNOCC. Given that each population of *V. shenzhenica* harbors unique genotypes, and the extinction of any population could result in a significant loss of genetic variation and genotype diversity, it is essential to relocate and conserve individuals from every population to ensure that the species’ genetic diversity is maintained. Since the distribution information of *V. shenzhenica* is still being updated, it is important to continuously identify new genotypes of additional populations and preserve them, thereby establishing a more comprehensive gene pool for *V. shenzhenica*. Regarding in situ conservation, it is crucial to minimize human activities that may impact wild populations. For this reason, tourism should be restricted within the distribution ranges of these populations. Additionally, relevant conservation authorities should conduct regular monitoring to assess the health and status of the populations and to identify potential risks. Secondly, for species that rely on clonal reproduction, it is important to accurately evaluate the effective population size using molecular approaches to guide conservation planning [9]. In this study, while the HZ population is recognized as the largest known population, spanning a distribution area of up to 1000 m^2^, it has been confirmed to be a monoclonal group with a single genotype. This indicates that regardless of the number of individuals, all ramets share an identical genetic profile derived from the same genet. By identifying the genotypes of various populations through genetic analysis, it becomes feasible to introduce different genotypes into distinct populations, thereby establishing a basis for genetic exchange and facilitating future sexual reproduction.

Lastly, in the long term, sexual reproduction remains a priority over clonal propagation due to its evolutionary advantages [43,44]. Species exhibiting prolonged clonal growth may not be as persistent due to a severe reduction in sexual recruitment and genotypic diversity [6]. However, the reproductive biology of *V. shenzhenica* remains poorly studied due to several challenges, including the low likelihood of simultaneous flowering across different groups in cultivation, the brief flowering period (a single flower typically lasts only a few hours after opening, which can be found in most *Vanilla* species [45], personal observation), and the significant distances between wild populations, all of which complicate related studies.

The population has not yet reached functional sexual extinction, as occasional flowering can still be observed in cultivated individuals and wild populations. Therefore, management plans should prioritize ex situ conservation efforts, with the primary goal of promoting assisted sexual reproduction to produce viable seeds and offspring that combine genotypes from different populations. This may require coordinated efforts between various institutions to prevent progression toward sexual extinction.

## 4. Materials and Methods

### 4.1. Taxon Sampling, Sequencing, SNP Calling, and Chloroplast Genome Assembly

Since *V. shenzhenica* was newly discovered in 2007 in Shenzhen, China, its distribution has not yet been fully understood. Aside from its type locality [11], this species has only been reported in Guangdong (Huizhou) [46], Hong Kong [47], and Fujian (Nanjing of Zhangzhou) [48]. Recently, it was also found in Vietnam [49]. In the present study, our team discovered three additional undocumented wild populations of *V. shenzhenica* through extensive field surveys in Guangdong, including one in Yangjiang and two in Guangzhou. To avoid the genotypic redundancy caused by clonal propagation, all foliar samples within a population were collected following a five-point spatial design—four peripheral points were positioned at the cardinal diagonals of the population’s distribution range and one central point, with a minimum 10 m spacing maintained between adjacent sampling loci (Table 3; Figure 1E); the population at the type locality which has since disappeared was excluded. Additionally, we sampled all available living individuals collected from wild populations found in Guangdong (Huizhou and Shenzhen) and transplanted at CNOCC for ex situ conservation purposes. All leaf samples (n = 43) were flash-frozen with liquid nitrogen and stored in EP tubes at −80 °C.

The genomic DNA of 43 samples was extracted using the HiPure SF Plant DNA Mini Kit (Magen). Qualified DNA was sent for ddRAD-seq library preparation using the ECORI (AATTC) and DPNII (GATC) enzyme combination, according to the protocol of Origingene Co., Ltd. (Shanghai, China). The final pooled libraries were sequenced on an Illumina NovaSeq 6000 platform with a paired-end 150 bp read length. Clean data were obtained after filtering the adapters and low-quality reads from the raw data using TRIMMOMATIC v.0.36 [50]. The de novo pipeline of STACKS v.2.54 [13], which includes the *ustacks*, *cstacks*, *tsv2bam*, *gstacks*, and *populations* modules, was applied for SNP calling. Subsequently, VCFtools v.0.1.13 [14] was used to filter the SNPs with the parameter set to “--maf 0.05 --max-missing 0.9 --mac 3”, i.e., the dataset includes only sites with a Minor Allele Frequency (MAF)—defined as the frequency of an allele across all individuals at a given site divided by the total number of non-missing alleles at that site—greater than or equal to 0.05. Additionally, the Minor Allele Count (MAC), which represents the total number of times the allele appears across all individuals at that site, must be at least 3. Sites with more than 10% missing data (i.e., with less than 90% non-missing data) are excluded.

This study assembled the *cp*DNA from the whole-genome skimming data, which were obtained from leaf samples, and paired-end sequencing libraries were constructed according to the Illumina library preparation protocol. Sequencing was carried out on an Illuminal NovaXplus platform at Origingene Co., Ltd. (Shanghai, China). All samples were sequenced to a target data point of 10 G. Clean data were obtained after quality filtering with Fastp v.0.23.4 [51]. *cp*DNA was assembled into two complete, reverse-oriented chloroplast genome sequences for each sample using GETORGANELLE v.1.7.7.1 [52]. After annotation in Geneious Prime 2024.0.5 [53], the sequences with the same direction as the reference genomes were selected for phylogenetic tree construction. The chloroplast genomes of *V. aphylla* (NC_035320) and *V. madagascariensis* (NC_046809) were downloaded from NCBI and were used as reference genomes for the annotation process.

### 4.2. Phylogenetic, Genetic Diversity, and Population Structure Analyses

To investigate the three undocumented populations of *V. shenzhenica* with living collections at CNOCC and to determine their relationship with the confirmed wild populations, a phylogenetic analysis based on *cp*DNA was conducted using the maximum likelihood (ML) method in IQ-TREE v.1.6.9 [54], as well as Bayesian inference (BI) analysis using MrBayes v.3.2.7 [55]. ModelFinder [56] was employed to determine the optimal nucleotide substitution model through Bayesian Information Criterion (BIC) evaluation. The selected best-fitting model was subsequently applied for ML tree reconstruction using IQ-TREE, with a setting of “-alrt 1000 -bb 1000”, to calculate the node support, combining the SH-aLRT test and ultrafast bootstrap with 1000 replicates. The Bayesian analysis using MrBayes implemented two independent runs of Markov chain Monte Carlo (MCMC) simulations (4 chains per run; 5 hundred thousand generations) with sampling every 1000 generations. Convergence was confirmed when the average standard deviation of split frequencies fell below 0.01. The complete *cp*DNA sequences of *V. aphylla* (NC_035320, LC_085348), *V. madagascariensis* (NC_046809, NC_200374), *V. planifolia* (MN_200375, NC_026778), and *V. somae* (NC_079955) were downloaded from NCBI and used as outgroups. The *cp*DNA sequence of *V. shenzhenica* in this study was submitted to NCBI (PQ_652344). The phylogenetic tree was visualized using ITOL v.6 [57].

The genetic diversity, including *H*_O_, *H*e, π, *F*_IS_, and *F*_ST_, was calculated using the *populations* procedure in STACKS v.2.54 [13] based on SNPs from the processing of ddRAD-seq data. The genetic relationships among populations, specifically genetic structure, were explored using three types of cluster methods. Bayesian assignment tests were performed on all individuals using ADMIXTURE v.1.3.0 [58], with the assumed number of substructures (*K*) set from 2 to 15. The optimal *K* value was determined based on the minimum cross-validation error, with the smallest value being the most likely to capture the major structure. PLINK v.1.90 [59] was used to conduct principal component analysis. The results of both the structure analysis and the PCA were visualized using the “barplot” function and “ggplot2” [60] package, respectively, in R version 4.3.3 [61]. To assess population isolation by distance, a Mantel test was performed to correlate pairwise genetic distance (*F*_ST_) and geographical distance (km) among populations based on SNPs, using the “ade4” [62] package with 9999 replicates in R version 4.3.3.

### 4.3. Demographic History

Based on SNP data, Stairway Plot v.2 [63] was used to infer changes in the effective population size (*N*e) between 1000 kya and 1 kya. Using the Python script EASYSFS v.0.0.1 [64], the variant call format (VCF) file containing SNP data was processed to construct a one-dimensional site frequency spectrum (SFS). The SFS was specified as folded, and the projection value that maximizes the number of loci was selected. The SFS information was then input into the blueprint file required for the Stairway Plot program. The mutation rate (u) per generation per site was set at 4.55 × 10^−9^, which was estimated using the formula u = Ks/2T [65,66], with a generation time of 4 years.

## 5. Conclusions

Based on the genome sequencing data from 43 *V. shenzhenica* individuals, we found that vegetative growth is the predominant mode of reproduction in all extant studied populations, consistent with the filed investigations. Moreover, all the wild populations were revealed to be monoclonal, each consisting of a unique single genotype. The analysis also indicated that a combination of declining effective population size, clonality, and environmental disturbances has contributed to the low genetic and genotype diversity of *V. shenzhenica*. In particular, the immediate ex situ preservation of all populations is necessary, as the extinction of any population could lead to a significant loss of existing genetic variation and genotype diversity. Management plans should prioritize ex situ conservation efforts, with the primary goal of promoting assisted sexual reproduction to produce viable seeds and offspring that combine genotypes from different populations, in order to prevent the loss of sexual reproduction. This may require joint efforts among various institutions.

## Figures and Tables

**Table 1:** Genetic diversity indices of different populations of *V. shenzhenica*.

Population	*H*o	*H*e	π	*F*_IS_
GZ_1	0.198	0.106	0.123	−0.131
GZ_2	0.215	0.113	0.127	−0.158
HK	0.205	0.109	0.121	−0.150
HZ	0.184	0.098	0.109	−0.134
YJ	0.210	0.165	0.185	−0.040
ZZ	0.172	0.142	0.159	−0.016
CNOCC_1	0.177	0.094	0.099	−0.148
CNOCC_2	0.219	0.114	0.136	−0.137
Species	0.214	0.135	0.148	−0.138

Note: *H*o, observed heterozygosity; *H*e, expected heterozygosity; π, nucleotide diversity; *F*_IS_, inbreeding coefficient.

**Table 2:** Pairwise fixation index (*F*_ST_) between each population of *V. shenzhenica*.

Population	GZ_1	GZ_2	HK	HZ	YJ	ZZ	CNOCC_1	CNOCC_2
GZ_1	0	0.430	0.437	0.467	0.327	0.380	0.455	0.471
GZ_2		0	0.420	0.449	0.316	0.372	0.445	0.452
HK			0	0.424	0.329	0.375	0.418	0.454
HZ				0	0.334	0.384	0.009	0.478
YJ					0	0.282	0.344	0.331
ZZ						0	0.388	0.375
CNOCC_1							0	0.470
CNOCC_2								0

**Table 3:** Sample information of *V. shenzhenica* for resequencing.

Population	Location	Altitude (m)	Sample Size	Voucher
GZ_1	Conghua, Guangzhou, Guangdong	460	4	JBC15371~JBC15374
GZ_2	Conghua, Guangzhou, Guangdong	356	5	JG12731~JG12735
HK	Hong Kong	75	5	JG12671~JG12672
HZ	Huiyang, Huizhou, Guangdong	181	5	JG12721~JG12725
YJ	Yangxi, Yangjiang, Guangdong	327	5	JG12691~JG12695
ZZ	Nanjing, Zhangzhou, Fujian	413	5	JG12741~JG12745
CNOCC_1	Living collections at CNOCC, potentially collected from Huizhou	-	11	C11~C13 D11~D13 D23, D24 D31~D3
CNOCC_2	Living collections at CNOCC, potentially collected from Shenzhen	-	3	D21, D22, D25
Total			43	

## Data Availability

The data presented in this study are available in the article.
